# Effectiveness and cost-effectiveness of a guideline-based stepped care model for patients with depression: study protocol of a cluster-randomized controlled trial in routine care

**DOI:** 10.1186/s12888-014-0230-y

**Published:** 2014-08-20

**Authors:** Birgit Watzke, Daniela Heddaeus, Maya Steinmann, Hans-Helmut König, Karl Wegscheider, Holger Schulz, Martin Härter

**Affiliations:** Department of Medical Psychology (W26), University Medical Center Hamburg-Eppendorf, Martinistraße 52, 20246 Hamburg, Germany; Clinical Psychology and Psychotherapy Research, Institute of Psychology, University of Zurich, Binzmühlestrasse 14/16, CH-8050 Zurich, Switzerland; Department of Health Economics and Health Services Research (W37), Hamburg Center for Health Economics, University Medical Center Hamburg-Eppendorf, Martinistraße 52, 20246 Hamburg, Germany; Department of Medical Biometry and Epidemiology (W34), University Medical Center Hamburg-Eppendorf, Martinistraße 52, 20246 Hamburg, Germany

**Keywords:** Depression, Stepped care, Collaborative care, Complex intervention, Primary care, Secondary care, Low intensity treatments, e-Mental health

## Abstract

**Background:**

Depression is a widespread and serious disease often accompanied by a high degree of suffering and burden of disease. The lack of integration between different care providers impedes guideline-based treatment. This constitutes substantial challenges for the health care system and also causes considerable direct and indirect costs. To face these challenges, the aim of this project is the implementation and evaluation of a guideline-based stepped care model for depressed patients with six treatment options of varying intensity and setting, including low-intensity treatments using innovative technologies.

**Methods/design:**

The study is a randomized controlled intervention trial of a consecutive sample of depressive patients from primary care assessed with a prospective survey at four time-standardized measurement points within one year. A cluster randomization at the level of participating primary care units divides the general practitioners into two groups. In the intervention group patients (n = 660) are treated within the stepped care approach in a multiprofessional network consisting of general practitioners, psychotherapists, psychiatrists and inpatient care facilities, whereas patients in the control condition (n = 200) receive routine care. The main research question concerns the effectiveness of the stepped-care model from baseline to t3 (12 months). Primary outcome is the change in depressive symptoms measured by the PHQ-9; secondary outcomes include response, remission and relapse, functional quality of life (SF-12 and EQ-5D-3 L), other clinical and psychosocial variables, direct and indirect costs, and the incremental cost-effectiveness ratio. Furthermore feasibility and acceptance of the overall model as well as of the separate treatment components are assessed.

**Discussion:**

This stepped care model integrates all primary and secondary health care providers involved in the treatment of depression; it elaborates innovative and evidence-based treatment elements, follows a stratified approach and is implemented in routine care as opposed to standardized conditions. In case of positive results, its sustainable implementation as a collaborative care model may significantly improve the health care situation of depressive patients as well as the interaction and care delivery of different care providers on various levels.

**Trial registration:**

This study is registered with ClinicalTrials.gov, number NCT01731717 (date of registration: 24 June 2013).

## Background

Depression is one of the most widespread mental disorders [1] and involves a high degree of personal suffering, a high burden of disease and serious impairments [2]. Thus it constitutes a substantial challenge for the health care system and causes considerable direct and indirect costs [3]. Detailed estimations predict a further increase in depression-related disease burden in the next 20 years; depression will represent the second most important factor for impairment and premature mortality after cardiovascular diseases in highly economically developed countries [2].

Health care is confronted with several central areas of concern in the diagnosis and treatment of depression:

1. Depression often remains undetected or is diagnosed late: Especially in the primary care setting, the current detection rate of 50% to 68% needs to be optimized [4–8].
2. Patients with depression often do not receive evidence-based treatments or the treatment is initiated only after a long delay [8,9]. Adults in urban areas have to wait 12.5 weeks on average for a first diagnostic contact, while in rural areas the waiting time for psychological treatments comprises up to 17 weeks, according to a national survey conducted by the German federal psychotherapist association [10]. Depressive patients on waiting lists suffer from a high burden of disease for many months [11].
3. The initiation of treatment for patients with depression is often carried out with a rather unsystematic selection related to the type, the intensity of treatment and the adequate allocation of resources. This may increase the risk of undersupply on the one hand and oversupply on the other hand [12]. In this respect, especially low-intensity treatment options (e.g. structured self-help approaches) systematically integrated into the health care system are missing.
4. When treatments are initiated, an integrated care spanning different sections of the health care system is complicated due to the fragmentation of health services and the absence of an appropriate interface management (e.g. between primary and secondary care and between out- and inpatient care) [13].

In order to overcome these deficits it is necessary to develop, implement and evaluate guideline-oriented and evidence-based models of care that include 1) better detection rates of depression, 2) the prompt offer and access to evidence-based treatments, 3) a systematic treatment selection with the option of choosing between different levels of treatment intensity to initiate a tailored and efficient treatment and 4) multi-professional cooperation of the providers across different health care sectors in order to ensure an integrated pathway through the health care system.

Stepped care is a promising approach for improved depression care [12,14,15], a concept recommended in national and international guidelines [16–18]. Stepped care is based on treating patients with the most adequate treatment of lowest intensity while continuously monitoring their treatment progress. If clinically necessary, patients are stepped up to a more intensive intervention form. The treatment level is adjusted gradually whenever indicated until a satisfactory health status is achieved [12]. Katon and colleagues [19–21] first demonstrated in the 1990s the effectiveness of several comprehensive models to improve the treatment of patients with depression in primary care: They found that a stepped care model consisting of psychoeducation and a higher frequency of psychiatric consultations led to increased medication compliance and reduced depressive symptoms in comparison to routine care, suggesting its effectiveness and cost-effectiveness [15,21]. Van’t Veer-Tazelaar et al. showed how a stepped care model (watchful waiting, bibliotherapy, short term psychotherapy and medication) for elderly patients with subclinical depression reduced the risk for the occurrence of a clinical depression by approximately 50% [22].

Taking these promising results into account, we developed and implemented an extended stepped care model into routine care integrating the following aspects:

1. Implementation of a guideline-oriented and evidence-based stepped care model within a multi-professional network across different health care sectors consisting of general practitioners, psychiatrists and psychotherapists in out- and inpatient care.
2. Systematic screening and structured diagnostic procedures in primary care as the basis for an appropriate delivery of different interventions following a stepped care approach.
3. Patients are treated within this network with the goal of delivering a prompt and appropriate evidence-based treatment with an improved information exchange between the involved providers.
4. Patients receive the treatment step which they are most likely to benefit from (as opposed to every patient receiving the least intense step first and being stepped up only if this step is not effective enough).
5. Introduction of evidence-based treatment options with different levels of intensity which are not yet available in German routine care. We include modern e-health interventions like Internet- and telephone-based psychotherapy as well as the more traditional approach of bibliotherapy in the stepped care model [17,23].

The aim of the randomized controlled trial is to evaluate this guideline-oriented and evidence-based stepped care model for depressive patients comprising six treatment options of varying intensity and setting, including innovative technologies. The evaluation refers to the effectiveness and the cost-effectiveness of this complex intervention under the conditions of routine care.

## Methods/design

### Setting

The study is embedded in the intersectoral research network *psychenet: Hamburg Network for Mental Health* (2011–2014), a research and development project funded by the German Federal Ministry for Education and Research (BMBF) [24]. It aims at the improvement of the city’s population’s mental health by implementing new integrated health care networks based on evidence for effective treatment methods and by evaluating selected innovations and complex interventions in the region of Hamburg. The overall project is organized in 11 sub-projects. While the first five sub-projects (1–5) facilitate mental health across different areas of disease by improving information and education, fostering occupational health or strengthening the participation of sufferers and their family members, five illness-specific health networks have been conceived (sub-projects 6–10). A general accompanying sub-project (11) ensures the quality of the interventions in all sub-projects and conducts evaluative and health-economic investigations [24]. This article describes the study protocol of the health network depression (sub-project 7).

### Objectives

Primary objective of this randomized controlled trial is to examine the effectiveness of a complex intervention (stepped care model, SCM) assessed through the reduction in depression severity within a network of general practitioners (GP), psychiatrists and psychotherapists in routine care.

Secondary objectives are to examine the effectiveness of SCM in terms of response, remission and health-related quality of life as well as to examine its cost-effectiveness. Further objectives are the evaluation of the process quality and of patients’ and practitioners’ satisfaction with SCM.

### Study design

The study is designed as a randomized controlled intervention trial of a consecutive sample of depressive patients from primary care assessed with a prospective multiple time point survey. GPs are divided into two groups by undertaking a cluster randomization on the level of the participating primary care units. Patients recruited by GPs in the intervention group (IG) are treated within the stepped care approach (SCM, see section *Intervention*). Patients recruited by GPs in the control group (CG) receive treatment as usual (TAU). Both patient groups are compared with respect to their treatment response within a one-year period. The scientific approach is primarily quantitative with additional qualitative analyses regarding feasibility and acceptance of the SCM by patients as well as by out- and inpatient care providers.

### Primary hypothesis

The trial evaluates the primary hypothesis that the SCM condition (IG) outperforms the TAU condition (CG) regarding the primary outcome parameter (PHQ-9), i.e. that stepped care is more effective than treatment as usual in depressive symptom reduction after 12 months.

### Randomization

Cluster-randomization is performed in order to allocate confounding variables in equal parts to both study conditions and thus to control for potential bias in order to guarantee internal validity. In this study, cluster-randomization is undertaken at the level of the participating GP practices, which are allocated to either IG or CG in a 3:1 ratio. The process of randomization is conducted by a computer program by minimization based on the GP’s practice size (single practice vs. group practice), the location of the practice within Hamburg classified into the two categories (central vs. peripheral) and income level of the practice’s local district classified into three categories (low, middle and high). Criteria for selection of clustering variables are relevance and availability. We assume that each GP in the 49 participating practices is able to recruit 15–25 patients. A total of 36 practices is randomized into the IG and 13 practices into the CG.

### Ethical approval

The study protocol was approved by the responsible local Ethics Committee in Hamburg and will be conducted according to the principles of the Declaration of Helsinki (2013 version).

### Study population and recruitment

#### Patients

**Figure 1:**
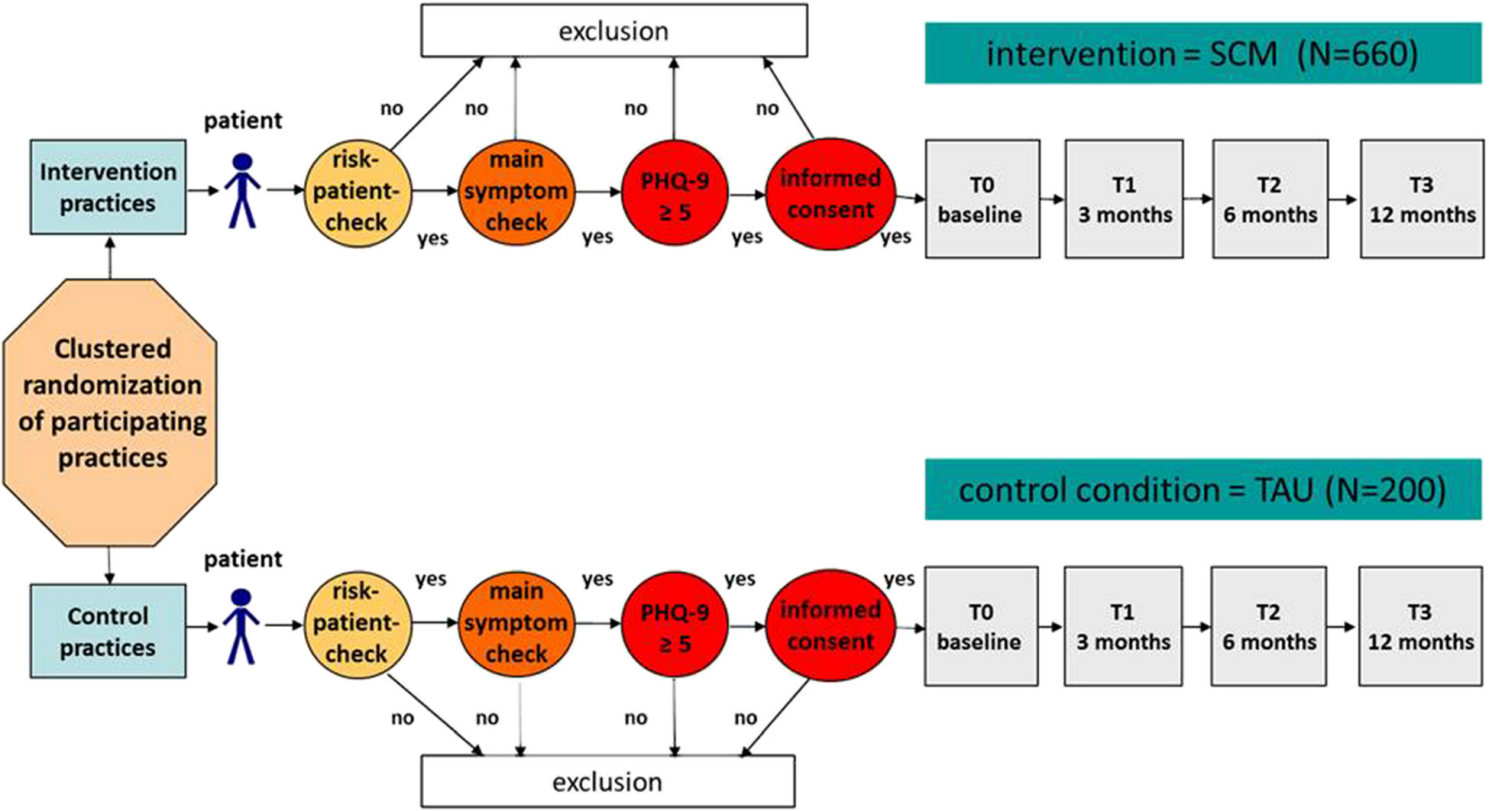
**Design of the study comparing a stepped care model (SCM) to treatment as usual (TAU) for patients with depression.**

The recruitment of the patients in the IG and CG is carried out by the participating GPs and comprises three guideline-recommended steps of screening and assessment as displayed in Figure 1: First, by applying a 2-item-checklist (*risk-checklist*) the GP systematically identifies those patients with diffuse somatic symptoms and/or with chronic somatic conditions [16,25], i.e. patients at high risk for depression. For this group of patients, the GP continues the screening procedure by using a further guideline-based 2-item-checklist (*main-symptom-checklist*) assessing the main symptoms of depression. A positive answer in at least one of these questions leads to the third screening step, an assessment with the depression module of the Patient Health Questionnaire (PHQ-9): Patients with a score of five or more points meet the study’s basic inclusion criterion and are informed about the study. After giving informed consent, patients receive their baseline questionnaire.

### Inclusion and exclusion criteria

Inclusion criteria are a minimum age of 18, five or more points on PHQ-9 and informed consent. Patients with insufficient knowledge of the German language or a health situation that does not allow questionnaire completion are excluded. Neither somatic nor mental comorbidities are exclusion criteria. However, if a mental disorder other than depression is the main treatment focus patients are also excluded from the study.

### Sample size and power calculation

We aim to gain a sample size that permits a small to moderate effect size between SCM and TAU to be detected with a statistical power of 80%. A small to moderate effect size is defined as eta^2^ = 0.0344 (or f = 0.175 according to Cohen [26]). We expect a reduction of the error variance of approximately 20% by including initial symptom severity (PHQ-9 at t0) as a covariate (which can be expected to be uncorrelated to the study conditions due to randomization). This leads to an adjusted effect size of eta^2^ = 0.042 (f = 0.209). In this respect and based on α = 0.05 a sample of n = 92 patients in each study condition is required for our main analysis. As we presume a drop-out rate of 20% in the IG an initial sample of n = 110 patients is needed in this group to answer the main research question. In order to run further separate analyses for each of the six treatment options within the SCM (and assuming an equal distribution of patients onto each option) we include 6*110 patients in the intervention group. As for the control group, a larger rate of drop-out has to be expected (approximately 50%, due to less personal involvement), a total of N = 200 patients is to be recruited in this study condition. Therefore, a total of N = 860 depressive patients are to be included in the study.

For the primary endpoint changes in PHQ-9 to baseline after 12 months we assume a small to moderate effect size defined as Cohen's d = 0.4 and set the significance level to 0.05 (two-sided). With these settings a sample size of 100 patients in each group is required to achieve a power of 80%. 2 × 100 patients need to be included if the randomization occurs at the patient level. With an assumed intra-cluster correlation (ICC) of 0.05 and 20 included patients per cluster on average a design effect of 1.95 is calculated, increasing the sample size to 200 patients and 10 practices per group.

We decide to recruit the threefold number of patients in the intervention group because we would like to obtain detailed knowledge about the six treatment options. And so we assume to have a sufficient number of patients in each of the treatment options. This approach results in 860 patients and 40 practices to recruit with a 3:1 allocation to the intervention and the control group, respectively.

### Intervention

#### Treatment condition

The SCM is embedded in a multiprofessional network (see below) and consists of the following components:

### Diagnostic process and treatment selection

GPs of the intervention group continue the diagnostic process with a paper-pencil ICD-10-checklist to determine depression type and severity. Patients are given psycho-education about depression including evidence-based patient information. The decision about which evidence-based treatment option to carry out is made in cooperation with the patient (shared decision-making). Treatment interventions are allocated with consideration to depression severity and patient preference following guideline recommendations. The six treatment options (as well as the step 1 option of watchful waiting), the rationale for treatment selection within stratified stepped care and the care provider responsible for each treatment option are displayed in Table 1. While these recommendations offer a rationale orientation for treatment decisions, the assessment of the individual’s preferences may lead to different individual treatment decisions.

**Table 1:** **Criteria for systematic treatment indication and description of responsible care providers for each step**

**Severity of the depressive disorder**	**Step in the SCM**	**Intervention**	**Responsible care provider**
Mild depressive disorder, duration: up to two weeks	1	Watchful waiting (active monitoring) for 2 weeks	GP
Mild depressive disorder, duration: over two weeks	2.a or 2.b	Bibliotherapy or Internet-based self-help program	GP
Mild to moderate depressive disorder, duration: over two weeks	2 plus	Telephone-based psychotherapy	Psychotherapist
Moderate depressive disorder	3.a or 3.b	Psychotherapy or pharmacotherapy	Psychotherapist or psychiatrist/GP
Severe depressive disorder without/ with suicidality	4	Combination therapy: psycho- and pharmacotherapy (inpatient or outpatient setting)	Psychotherapist and psychiatrist; clinic

### Step 2.a/2.b/2 plus

Three forms of low-intensity treatment for patients with mild depressive disorders are offered: 1) step 2.a: bibliotherapy; 2) step 2.b: the Internet-based self-management program “deprexis®” and 3) step 2 plus: telephone-based psychotherapy with a previous face-to-face contact conducted by a licensed psychotherapist.

In bibliotherapy, patients work independently and at their own pace with a cognitive-behavioral self-help book (“Selbsthilfe bei Depressionen”) [27], comprising detailed psycho-education and exercises with a focus on behavioral activation and cognitive restructuring. Personal guidance is limited in its scope and is carried out by the GP who gives an initial introduction to the intervention, hands out the book and monitors the progress of patients in this step.

In Internet-based self-management, the GP informs the patient about the program and the procedures and provides the patient with a personal license to register to the software. The program deprexis® is a certified medical product based on a cognitive-behavioral approach that allows patients to work independently and at their own pace [28]. The program consists of 12 interactive simulated conversations including detailed psycho-education and exercises. It focuses on behavioral activation and cognitive restructuring. For each patient, the program is tailored individually in terms of level of detail, language and personal relevance. The GP monitors and accompanies the patient during the three months scheduled to complete the program.

The telephone-based psychotherapy is the translated and adapted version of a specific intervention program [29–31] developed by researchers in Seattle. It includes a patient workbook as well as a therapist manual and will be evaluated in two conditions (delivery with vs. without additional motivating letters from the psychotherapist after each session). It comprises 8 to 12 telephone contacts (20 to 40 minutes) which are carried out weekly and - at a later treatment stage - biweekly. This 3-month structured program also follows a cognitive behavioral approach with the main focus on behavioral activation and cognitive restructuring. As the level of guidance and intensity in telephone-based psychotherapy is higher than in bibliotherapy and Internet-based self-management, it can also be carried out with patients suffering from moderate depression, especially if they decline step 3 (psychotherapeutic or pharmacological treatment).

### Step 3 and Step 4

Psychotherapy and/or pharmacotherapy (as stand-alone and as combination treatment) are conducted by health care providers in outpatient care (psychotherapy: psychologists or physicians licensed as psychotherapists; pharmacotherapy: psychiatrists or GPs) from routine care who undergo initial training and take part in four quality circles per year (see next section).

### Monitoring

Depression severity is systematically monitored by the responsible care provider within defined time intervals in order to ensure that a potential under- or oversupply is detected as quickly as possible. During monitoring patients fill out the PHQ-9 which is checked by the provider. Additionally, the care provider (GP, psychiatrist or psychotherapist) completes a monitoring sheet. This checklist assesses treatment-related information and is designed to facilitate decisions regarding further treatment (continuing current treatment, stepping up or down, treatment termination).

### Multiprofessional network

A necessary framework for the SCM is its integration in a network consisting of the relevant care providers involved in the treatment of depressive patients, i.e. GPs, psychotherapist, psychiatrists and inpatient care facilities. To build such a network these professional groups were sent information and personal invitations to join the project. Additionally, articles in professional journals advertising the study were published and professionals were contacted via telephone.

The main focus of the network is facilitation and enhancement of information exchange and communication between all network care providers of the network in order to increase the quality of patients’ health care. Another important aspect is the prompt referral to a secondary care provider like psychotherapist or psychiatrist. Information exchange about available treatment capacities in secondary care is enhanced using an online tool specifically developed for this project. Psychotherapists and psychiatrists indicate whether they currently have an available treatment capacity which implies that the psychiatrist or psychotherapist is able to offer the patient a first contact within the next three weeks. This way, GPs are able to make a reservation for their patients online and refer them into secondary treatment without delay.

### Training and quality standards

Previous to the implementation of the SCM, participating care providers obtain training regarding the recommendations of the German National Clinical Practice Guideline for unipolar depression [18], the rationale and the treatment concept of the SCM and the specific interventions. Each GP is additionally visited by the study team at least once in his or her practice to review the diagnostic routines in the everyday routine care. The psychotherapist for the telephone-based treatment receives special training and weekly supervision. Within the network, guideline-based quality standards are defined (e.g. type and frequency of monitoring). To ensure that these standards are met and to promote the cooperation and information exchange between the participating care providers, quarter-yearly quality circles take place. For the conception of the initial training and quality circles we could integrate experiences from former projects [32–35].

### Intervention for the control group

Patients in the control condition receive treatment as usual by their GP and within the regular German health care system. However, systematic screening with the risk-checklist, main-symptom-checklist and PHQ-9 are carried out in both intervention and control group to ensure a comparable recruitment and inclusion process.

### Outcome assessment

The main data collection comprises paper-pencil questionnaires which are filled out by the patients at four time points: The baseline questionnaire (t0) is handed out by the GP at intake and filled out before any treatment begins. The questionnaires 3 months (t1), 6 months (t2) and 12 months (t3) after baseline are sent to the patient’s by mail. For each completed questionnaire, patients receive an incentive of 5 €. Patients who fail to send back their questionnaires are reminded to do so twice by mail.

The *primary outcome parameter* for effectiveness is the change in depressive symptoms from baseline to t3 assessed by the PHQ-9. *Secondary outcome parameters* are response (defined as a 50% reduction in the PHQ-9 from t0 to t3) and remission (defined as < 5 points in the PHQ-9 at t3), change in health related quality of life (SF-12 and EQ-5D-3 L) and further clinical and psycho-social variables. To evaluate the cost-effectiveness of the SCM, direct and indirect costs as well as quality-adjusted life years (QALYs) based on the EQ-5D index are measured and the incremental cost-effectiveness ratio (ICER) is computed as further secondary outcomes.

Process analyses refer to treatment paths, decision processes and interface management. Additionally, feasibility and acceptance of the overall model of stepped care and of its components are analyzed taking patient and care provider perspectives into account. Table 2 summarizes the instruments for the patient self-ratings.

**Table 2:** **Instruments and measurement points (patient self-ratings)**

	**Measurements**			
**Outcome variables**	**T0**	**T1**	**T2**	**T3**
Patient Health Questionnaire (PHQ-D)	X	X	X	X
Short Form Health Survey (SF-12)	X	X	X	X
EuroQol (EQ-5D-3 L)	X	X	X	X
General self-esteem scale (Rosenberg Self-Esteem-Scale RSES)	X	X	X	X
General self-efficacy scale (GSE)	X	X	X	X
Self-efficacy for management and relapse prevention in depression	X	X	X	X
Depression self-management behavior	X	X	X	X
Medical treatments and services received during the last 6 months	X	-	X	X
Medication during the last 6 months	X	-	X	X
Health care utilization during the last 3 resp. 6 months	-	X	X	X
Satisfaction with specific treatments during the last 3 resp. 6 months	-	X	X	X
Client Satisfaction Questionnaire (CSQ-8)	-	-	-	X
Helping Alliance Questionnaire (HAQ)	-	X	X	X
**Further (controlling) variables**	**T0**	**T1**	**T2**	**T3**
Sociodemographics	X	-	-	-
Social Support (F-Sozu-14)	X	-	-	-
Shared decision-making (SDM-Q-9)	X	-	-	-
Treatment motivation (FPTM-23, subscales “psychological burden” and “expectations”)	X	-	-	-
Former depression-specific treatments	X	-	-	-
Symptom course of depression	X	-	-	-
Referral procedures and interface management	-	X	X	X
Self-help experiences and habits	X	-	-	-

### Outcome instruments (patient ratings)

#### Patient Health Questionnaire (PHQ)

The German version [36,37] of the Patient Health Questionnaire [38] is used. Specific subscales assessing following syndromes were selected: major depressive syndrome (9 items), generalized anxiety syndrome (7 items), somatoform syndrome (13 items) and panic syndrome (11 items). Additionally, psychosocial functioning resp. psychosocial stressors are measured.

### Short Form Health Survey (SF-12)

The SF-12 assesses overall health-related quality of life and is based on the Short Form 36 Health Survey [39]. It is composed of the two subscales physical health and mental health. Scores are computed by calculating the sum of the 12 weighted items and then transforming it to a scale from 0 to 100, on which high scores indicate a high level of health-related quality of life.

### EQ-5D-3 L

This generic self-rating instrument measures health-related quality of life on five dimensions: mobility, self-care, usual activities, pain/discomfort and anxiety/depression [40,41]. Each dimension has three response categories that represent three levels of severity (“no problems”/“some or moderate problems”/“extreme problems”). Additionally, a visual analogue scale allows the general assessment of health-related quality of life. From the EQ-5D-3 L a preference-based index of health-related quality of life (EQ-5D index) can be derived [42].

### General self-esteem scale (revised version of the Rosenberg Self-Esteem-Scale)

The scale was originally designed by Rosenberg in 1965 to assess global self-esteem and translated to German [43]. It contains 5 positively and 5 negatively formulated items with statements referring to the subject’s global attitude towards him- or herself. The answers are given on a four-point scale. After recoding negative items a total score can be computed.

### General self-efficacy scale (GSE)

This self-rating instrument measures global optimistic beliefs about one’s self on a one-dimensional scale [44]. The underlying concept of self-efficacy includes the self-related expectation to be able to successfully cope with difficult situations. On ten items formulated as statements, subjects indicate their level of agreement on a four-point scale.

### Self-efficacy for managing and preventing depression

The depression-related self-efficacy is assessed on a ten-point scale, where subjects indicate the extent of trust in their own ability to cope with their depressive symptoms and complaints [45]. Psychometric analyses have confirmed the scale’s internal consistency.

### Depression-related self-management behavior

These five items cover depression-related self-management behavior to assess patients’ behavioral strategies for handling depression [46]. Patients are questioned about the frequency with which they integrate pleasant and social activities into everyday life as well as about their attention regarding depressive symptoms, early warning signs and situations which put them at risk of depressive episodes.

### Health care utilization during the last 3 resp. 6 months, satisfaction with specific treatments during the last 3 resp. 6 months

These two parts of the questionnaire aim to explore which offers patients made use of during the last 3 respectively 6 months and how they are perceived and evaluated.

### Client Satisfaction Questionnaire (CSQ-8) (German version ZUF-8)

On eight items, this questionnaire measures the extent of the patient’s satisfaction with received medical and psychotherapeutic treatment [47,48]. Important aspects are the interaction between care provider and patient, information about received treatment elements and the communication between the care providers.

### Helping Alliance Questionnaire (HAQ)

The German short version of this questionnaire consists of 11 items rated on a six-point scale [49]. The HAQ assesses the two factors “perceived helpfulness” and “collaboration or bonding”. It aims to capture the patient’s view of the relationship with the psychotherapist and of process variables. High scores indicate a high level of relationship quality.

### Additional control variables (patient-ratings)

#### Socio-demographic data

Following socio-demographic data is collected within a structured questionnaire: age, gender, nationality, family status, partnership, children, educational background, vocational training, housing and professional situation.

### Social Support (F-Sozu-14)

The 14-item Social Support questionnaire assesses perceived social support (practical and emotional support). By computing means, a total score from 1 to 5 points can be calculated [50].

### Shared decision-making (SDM-Q-9)

This instrument measures the extent to which the patient is involved in the decision-making process for the treatment [51]. It consists of nine items rated on a 6-point scale.

### Treatment motivation: specific subscales of the German Psychotherapy Motivation Questionnaire (Fragebogen für Psychotherapiemotivation FPTM-23)

To explore the different aspects of patients’ self-rated motivation for psychotherapeutic treatment the short version of this instrument with 23 items rated on a 4-point scale is employed [52].

### Further self-developed variables

Depression-specific treatments received prior to the study, medical treatments and services received during the last 6 months and medication during the last 6 months, symptom course of depression, referral procedures and interface management, self-help experience and habits.

### Additional instruments (health care provider ratings)

#### Documentation forms for screening, diagnostic and monitoring

These forms help to facilitate and support the diagnostic and treatment process (and are therefore elements of the intervention itself), as well as to document clinical processes for research purpose: They are used to analyze clinical treatment pathways and their quality as well as to draw conclusions about care providers’ adherence to the guidelines.

### Ratings on feasibility and acceptance

Each health care provider in the SCM fills out a questionnaire assessing the feasibility of and satisfaction and experiences with the SCM as a whole, the single components of the SCM (especially the innovative elements) and the quality of the multi-professional network.

### Structural information

Information regarding structural and organizational aspects of the primary care practices and the GPs are gathered in an interview at the beginning of the study. Issues covered are the size of the practices as well as their equipment, catchment area, workload and proportions of different work aspects (patient contacts, administrative tasks, etc.).

### Statistical analyses

The primary analysis of the change in depressive symptoms from baseline to t3 assessed by the PHQ-9 will be based on the ITT-population. In case of missing follow-up values, a last-observation carried forward (LOCF) imputation will be performed, that is, the baseline determination will be imputed as follow-up determination. A linear mixed model will be calculated with group (SCM/TAU) as a fixed effect and practice as a random effect under control of the baseline value of the PHQ-9 as covariate.

Only the result of this primary efficacy analysis will be interpreted in a confirmatory manner.

The secondary endpoints will be examined in an exploratory manner with appropriate procedures, including subgroup analysis of sex, socio-economic status and symptom severity of patients. Analyses of secondary endpoints should provide an indication on the consistency of the results from the evaluation of primary endpoints. The effects of the selected strata in the minimization algorithm on the primary and secondary endpoints will be evaluated additionally. Regression coefficients, 95% confidence intervals and p values will be reported. The analyses will be conducted with the newest SPSS version.

With regard to the assessment of cost-effectiveness direct and indirect costs will be calculated. Administrative and market prices will be used to value resource utilization. The human capital approach will be employed to value productivity losses. As effect measure QALYs will be calculated from the EQ-5D-3 L. The cost-effectiveness analysis will be performed from a societal perspective. As point estimate of cost-effectiveness the ICER (incremental costs per QALY) will be calculated. To assess the uncertainty of the results a cost-effectiveness-acceptability-curve (CEAC) based on non-parametric bootstrapping will be computed. A net-monetary benefit regression analysis will be performed.

## Discussion

This randomized-controlled intervention study investigates the effectiveness and efficacy of a stepped-care model for patients suffering from depression. The model’s aim is to offer adequate and integrated care by providing six different intervention options of varying intensity levels and by implementing the recommendations of the German National Clinical Practice Guideline for unipolar depression.

A strength of the study is that the stepped-care model under investigation integrates the professional providers of primary and secondary care involved in the treatment of depression (GPs, psychiatrists and psychotherapists in out- and inpatient units) within one network in order to optimize treatment paths. Evaluating new innovative treatment elements and implementing the SCM in routine care - as opposed to standardized “ideal” conditions - are two further important characteristics of this study. The evaluation of the former aspect investigates whether these innovative treatments could represent worthwhile expansions to the German health care system for patients with depression. The latter aspect addresses the question of generalizability and transferability of findings to routine care.

Implementing the complex intervention of SCM into everyday clinical practice (with all its restrictions concerning time, resources, motivation etc.) cannot be carried out in as standardized a manner as it would be in settings more prone to research. However, process variables are assessed in order to ensure treatment adherence to SCM within our study. A further limitation is that we will not be able to make inferences about the effectiveness of specific elements of stepped care, as our design refers to the effectiveness of stepped care as a complex intervention.

In contrast to other studies, the SCM examined here follows a *stratified* stepped-care approach taking into account patients’ needs and preferences which may be clinically more adequate than the more stringent model of stepped care which begins with the least intensive treatment for each patient regardless of symptom severity. Together with the systematic monitoring aimed at ensuring an adequate treatment modality and dose, these innovative aspects can provide important findings about how stepped care should be designed to gain sizeable effects.
